# Broadband asymmetric light transmission through tapered metallic gratings at visible frequencies

**DOI:** 10.1038/srep39166

**Published:** 2016-12-13

**Authors:** Bin Tang, Zhongyang Li, Zizhuo Liu, Francois Callewaert, Koray Aydin

**Affiliations:** 1 School of Mathematics & Physics, Changzhou University, Changzhou 213164, China; 2 Department of Electrical Engineering and Computer Science, Northwestern University, IL 60208, USA

## Abstract

Asymmetric transmission phenomenon has attracted tremendous research interest due to its potential applications in integrated photonic systems. Broadband asymmetric transmission (BAT) is a highly desirable but challenging functionality to achieve in the visible regime due to the limitation of material dispersion. In this paper, we propose and numerically demonstrate that a tapered-metal-grating structure (TMGS) can achieve high-contrast BAT spectra covering the entire visible region. The transmission efficiency reaches ~95% for the forward illumination and ~35% for the backward illumination at the same wavelengths, respectively, and the corresponding transmission ratio is larger than 2.5 over a broadband wavelength regime. Such a design with high performance suggests applications for unidirectional optical transmission, optical diode, and so on.

Asymmetric transmission, referring to the transmission difference between forward and backward illumination through an optical device, has been an active research topic due to its potential applications in integrated photonic systems for all-optical computing and information processing^1^,^2^,^3^, such as optical interconnection and multiplexing^4^. A conventional way to achieve asymmetric transmission of light can be obtained by using magneto-optical media that possess an anti-symmetric off-diagonal permittivity tensor. The representative example is the application of optical isolators^5^, in which the Lorentz reciprocity is broken in the presence of an external magnetic field. Alternatively, it has been shown that one can completely reproduce the magnetic-optical effect by spatiotemporal modulation^6^,^7^,^8^. Meanwhile, wide varieties of materials have been employed in the design of optical diodes, such as anisotropic^9^, left-handed^10^, nonlinear^11^ or random amplified materials^12^. Also, asymmetric transmission can be realized in different engineered material systems such as composite grating structures^13^,^14^,^15^,^16^,^17^,^18^,^19^, photonic crystals^20^,^21^, metal-silicon waveguides^22^, and even in composition-graded semiconductor nanowires^23^. Recently, theoretical and experimental studies have proven that chiral metamaterials^24^,^25^,^26^,^27^,^28^,^29^, hyperbolic metamaterials^30^ and all-dielectric digital metasurfaces^31^,^32^ can enable asymmetric transmission for circularly and linearly polarized light^29^,^33^. By judiciously engineering parameters of individual building blocks, such as geometry, size and material, metasurfaces are promising to replace conventional electromagnetic elements in nanophotonic devices^34^,^35^. However, to the best of our knowledge, few works were concerned with the broadband asymmetric transmission (BAT) in the visible frequency range, and most of the proposed diode-like devices with asymmetric transmission were designed to operate either in a narrow band^30^ or beyond the visible spectrum range^36^.

In this paper, we propose and numerically demonstrate a simple but elegant design composing of a tapered-metal-grating structure (TMGS) that can achieve a BAT with high transmission contrast between forward and backward illuminations at visible frequencies. The maximum transmission efficiency reaches ~95% for the forward illumination and ~35% for the backward illumination, respectively, and the corresponding transmission ratio is larger than 2.5 over a broadband wavelength region. The operation wavelength in our proposed TMGS covers the entire visible frequency by optimizing relevant parameters such as grating period, substrate refractive index, and metal thickness. The results obtained in this paper provide intriguing possibilities to the design of optical devices based on broadband diode-like asymmetric transmission.

## Results and Discussions

**Figure 1:**
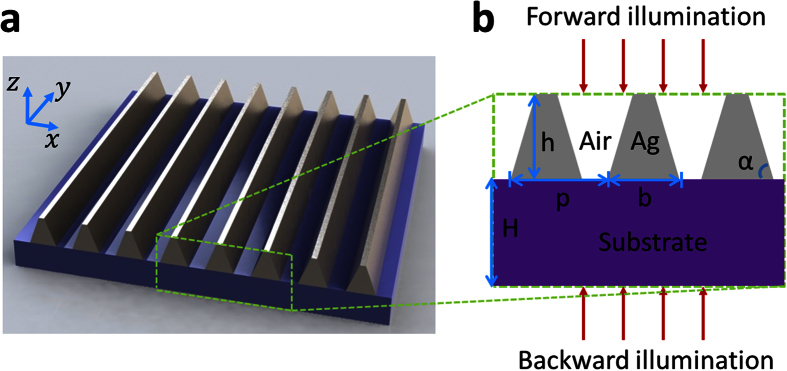
(**a**) Schematics of a tapered-metal-grating structure (TMGS) on the transparent substrate and (**b**) the corresponding cross section.

Figure 1(a) illustrates the schematic of the designed TMGS on top of a transparent substrate (sapphire). The metal (Ag) grating period is *p*, and the tapered ridges are characterized by the bottom span *b*, grating thickness *h* and angle α, which are shown in Fig. 1(b). The slit opening in the TMGS decreases from top to bottom. The finite difference time domain (FDTD) method is employed to model the electromagnetic response of the structure for a linearly polarized TM plane-wave excitation. In calculations, the periodic boundary condition is set in the *x*-direction and perfectly matched layer is used in the *z*-direction. The structure is assumed to be infinitely long in the *y*-direction and all simulation results have been normalized to the incident light power. The complex refractive index of metal Ag is chosen from the data of Palik (0~2 μm)^37^.

**Figure 2:**
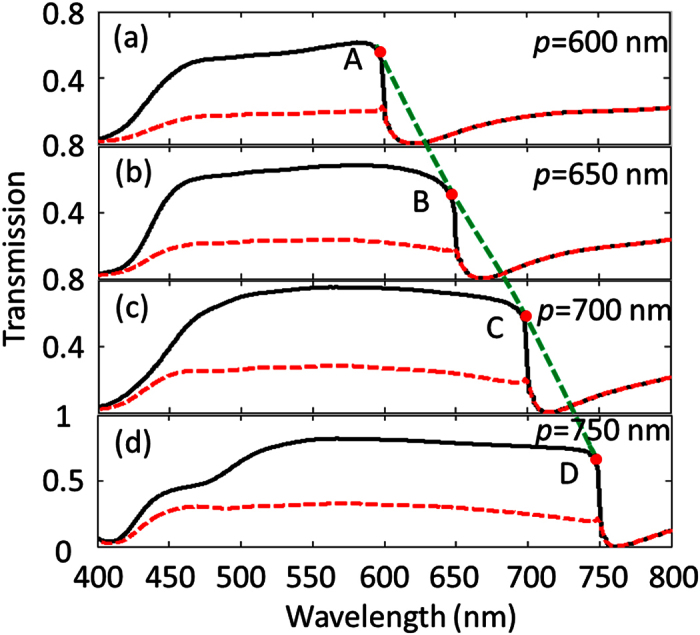
Transmission spectra of light through air-suspended TMGS for different periods *p* for the forward illumination (denoted by black solid line) and backward illumination (denoted by red dashed line). (**a)** *p* = 600 nm, (**b**) *p* = 650 nm, (**c**) *p* = 700 nm, and (**d**) *p* = 750 nm. Point *A*, point *B*, point *C* and point *D* are the positions of Wood-Rayleigh anomaly, respectively.

Using FDTD, we simulated the transmission spectra through air-suspended TMGS with different periods and for normal incident light propagation, approaching the structure from the top (forward) as well as from the back-side (backward). Simulations are performed using the following grating parameter values: *b* = 500 nm, *α* = 68°, and *h* = 500 nm. Figure 2(a–d) plot simulated transmission spectra for TMGS for four different periodicities (*p* = 600 nm, 650 nm, 700 nm, 750 nm) for forward (black solid line) and backward (red dashed line) illumination. It is clear in Fig. 2 that the transmission intensity depends significantly on the propagation direction for such a tapered grating structure. The bandwidth of the asymmetric transmission spectrum depends strongly on the periodicity of the grating *p*, and there exists a cut-off wavelength corresponding to the Wood-Rayleigh anomaly wavelength^38^ given by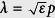, where *ε* is the permittivity of the substrate. The cut-off wavelength in Fig. 2(a–d) (labeled by point *A, B, C*, and *D*, separately) is approximately equal to the period considering of the substrate being air (*ε* = 1). Cut-off point red-shifts for increased periodicities as one would expect from the equation. Our numerical simulations are in good agreement with theoretical predictions. It is worthy of note that, Wood-Rayleigh anomaly is not a resonant phenomenon and the position merely depends on the periodicity of the grating and the refractive index of the surrounding medium.

**Figure 3:**
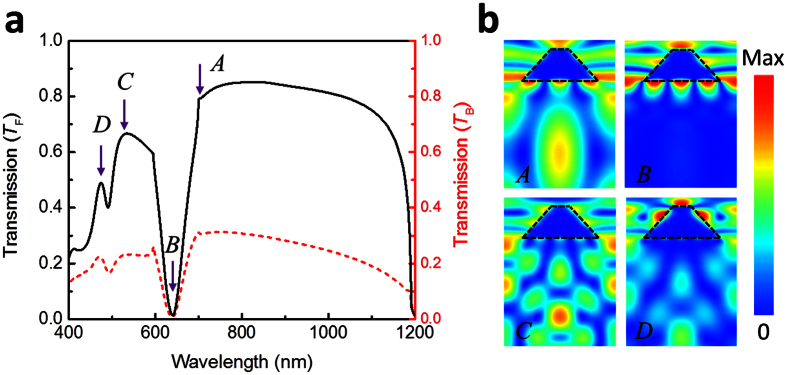
(**a**) Transmission spectra for different illumination direction. The black solid curve represents the forward illumination, and the dashed line denotes the backward illumination. (**b**) The distribution of magnetic magnitude (*H*) at different resonant wavelengths. The resonant wavelength at point *A* is 700 nm, point *B* is 632 nm, point *C* is 525 nm, and point *D* is 458 nm.

In the above discussion, we utilized a simple design composed of periodic metallic grating without any nonlinear media and complex geometrical shapes such as chiral meta-molecules to enable asymmetric transmission. Also the refractive-index profile is symmetric for both sides of the gratings, since initial simulations are performed for air-suspended TMGS. However, for practical purposes, realizing air-suspended metallic gratings is rather challenging, therefore simulation results for TMGS on a substrate is plotted in Fig. 3. As a substrate, we used sapphire with a constant refractive index of *n* = 1.7 in numerical simulations. As expected, it is still possible to achieve direction-dependent transmission characteristics even if the presence of a substrate with a higher refractive index. Comparing with the air-suspended TMGS, the Wood-Rayleigh anomaly wavelength in Fig. 2 (λ = 700 nm) disappeared, and on the contrary, it turned into a resonant transmission peak. The new position of anomaly wavelength has a red shift, and it can be calculated by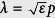 = 1.7 × 700 = 1190 nm, which coincides well with the numerical simulation result. Specially, the asymmetric transmission window is extended to the near-IR wavelengths, and multiple asymmetric transmission bands come to appear due to excitation of higher order modes with the increasing of metal grating thickness. To clearly prove this point, we calculated the distribution of magnetic field magnitude (*H*) at different resonant wavelengths (e.g. *A, B, C, D*) as shown in Fig. 3(a). One can see from Fig. 3(b), there exists a fundamental mode inside the dielectric substrate at point *A*. Meanwhile, there appear some higher modes that are excited due to the multiple resonant effects.

**Figure 4:**
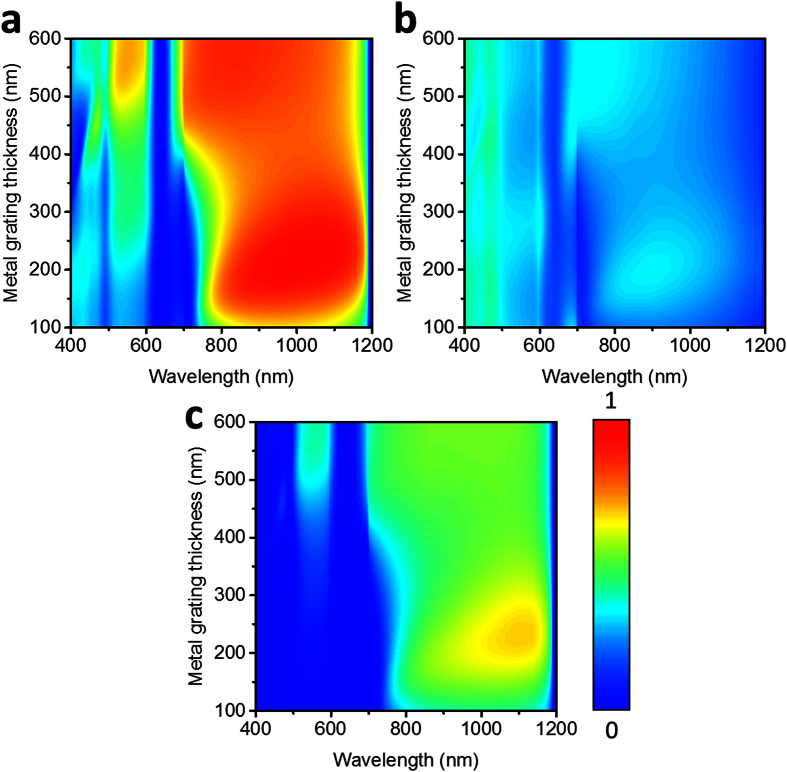
Bidirectional transmission spectra of light through the TMGS with a sapphire substrate versus wavelength for normal incidence and metal grating thickness of TMGS with fixed periodicity *p* = 700 nm, and the other parameters keep the same as Fig. 2. (**a**) forward illumination, (**b**) backward illumination, and (**c**) transmission difference between forward and backward illumination.

To deeply understand the effects of main parameters on the asymmetric transmission performance in the presence of substrate, additional electromagnetic simulations were performed based on the FDTD method. We firstly plotted the transmission spectra for forward and backward propagation directions from TMGS as a function of metal thickness varying from 100 nm to 600 nm and wavelength of light in Fig. 4, respectively. Similar calculations for air-suspended TMGS can be found in Supplementary Figure S1 (see Supplementary Information online). In these simulations, the periodicity is fixed at *p* = 700 nm, other parameters were kept the same as Fig. 2. It is clear that the metal thickness plays a significant role in determining the transmission intensity especially for forward propagation direction, therefore affecting the asymmetric transmission behavior drastically. For thinner metallic gratings, there is no noticeable difference in transmission intensity between the forward and backward propagation at the visible frequencies.

**Figure 5:**
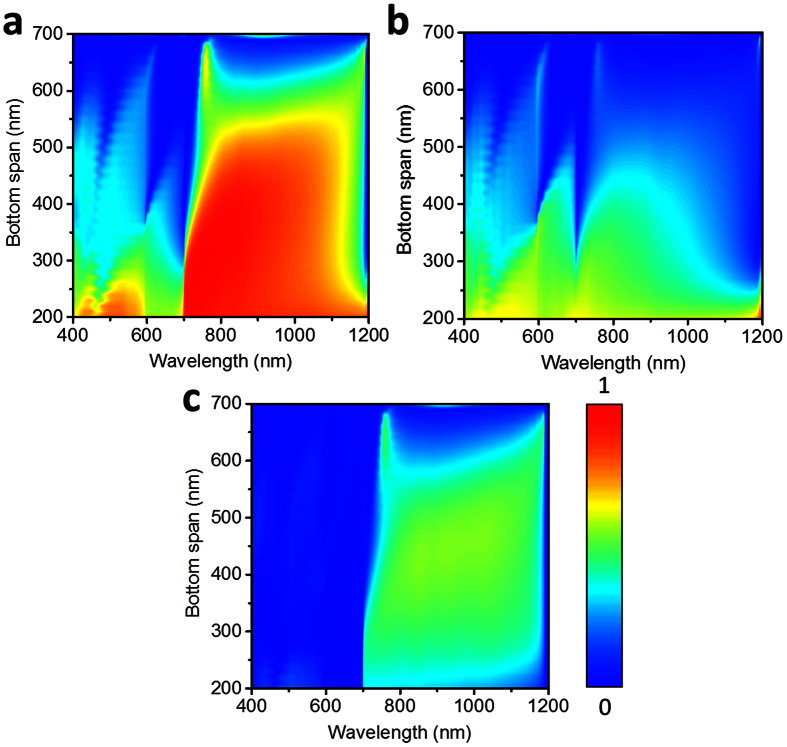
Bidirectional transmission spectra of light through the TMGS with a sapphire substrate versus wavelength for normal incidence and bottom span of metal grating with *h* = 500 nm, and the other parameters keep the same as Fig. 4. (**a**) forward illumination, (**b**) backward illumination, and (**c**) transmission difference between forward and backward illumination.

Meanwhile, we noticed that the bottom span *b* and angle α, which are important parameters in the building block of TMGS as shown in Fig. 1(b), have an obvious effect on the asymmetric transmission when the light is coming from two opposite directions. Figure 5 depicted the bidirectional transmission spectra of light through the TMGS with a sapphire substrate versus wavelength for normal incidence and bottom span of metal grating with *h* = 500 nm, and the other parameters keep the same as Fig. 4. Also, the difference between two different illuminations was shown in Fig. 5c to show the results more directly. From this picture, one can observe that bottom span *b* of metal grating also has an important effect on the asymmetric transmission, which mainly happens on the near-IR band. Further calculations and discussions on the effect of the angle α of TMGS can be found in Supplementary Figure S2 (See Supplementary Information online).

**Figure 6:**
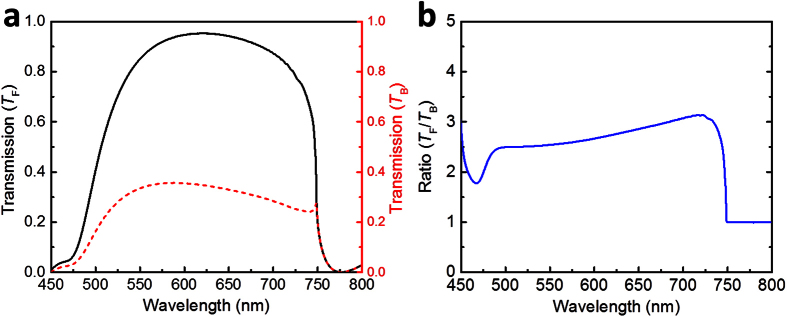
(**a**) Bidirectional transmission spectra of light through an optimized TMGS deposited on the sapphire dielectric. The main parameters take the following values: the period is *p* = 440 nm, and bottom span *b* = 280 nm, *h* = 120 nm, *α* = 68°. (**b**) The ratio of the forward and backward transmission spectra.

According to the above discussions, next we further optimized the grating parameters to enable broad transmission window at visible frequencies. Optimized parameters utilized in the simulations are *p* = 440 nm, *b* = 280 nm, *h* = 120 nm, and the angle is kept the same *α* = 68°. Figure 6(a) plots the transmission spectra of light propagating through an optimized TMGS on a sapphire substrate with forward (*T*_F_) and backward (*T*_B_) illumination. Figure 6(b) shows the ratio of transmission intensity, that is, *T*_F_ is divided by *T*_B_. From Fig. 6(a), one can see that there exists an ultra-wide asymmetric transmission band covering the entire visible region, in which the maximum transmission efficiency reaches ~95% for the forward illumination and ~35% for the backward illumination, respectively. The corresponding transmission contrast is larger than 2.5 in a broadband wavelength regime as shown in Fig. 6(b). The contrast can be increased even further using thicker metallic gratings. Here, the period *p* = 440 nm is chosen for optimum broadband transmission for forward propagation direction at visible frequencies. Although including a transparent substrate below our tapered metallic grating arrays affect the spectral position of the transmission window, asymmetric transmission effect can be still obtained by changing grating parameters accordingly. Additional simulations discussing the effect of the refractive index substrate on the transmission spectra are included in Supplementary Figure S3 (See Supplementary Information online).

**Figure 7:**
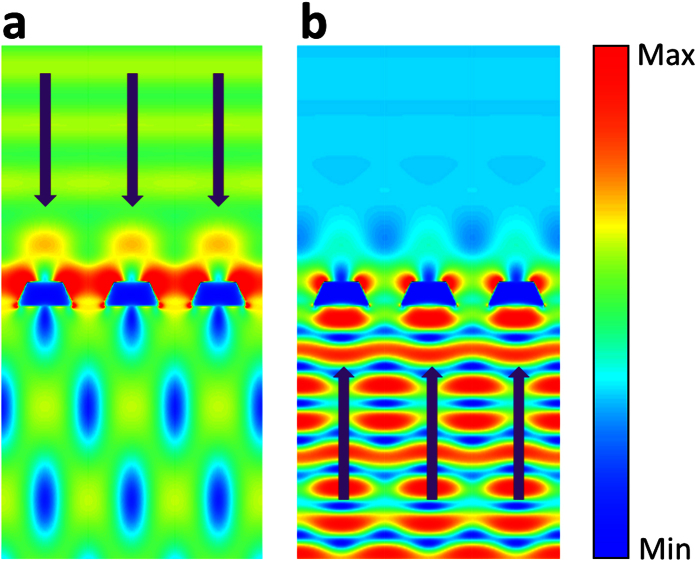
The profiles of electric field intensity for (**a**) forward illumination and (**b**) backward illumination at the wavelength 600 nm.

It is seemingly counter-intuitive for a metallic grating to enable asymmetric transmission, especially when there are no non-linear effects and complex geometrical shapes are included in the design. To gain more insights into the BAT phenomenon in the presence of sapphire substrate, we calculated the electric field density distribution throughout the TMGS for forward and backward illuminations. The profiles of electric field intensity at the wavelength 600 nm are shown for forward (Fig. 7a) and backward (Fig. 7b) propagation directions. From Fig. 7(a), we can clearly see that localized SPPs are excited at the corner of the trapezoidal cross-section under the forward illumination of TM polarized light due to the grating effect. The constructive interaction of both SPPs waves on slit walls enhances the radiation transmission for the case of forward illumination. On the contrary, for the backward illumination, most of the incident light is reflected back from the bottom-section of TMGS which is much wider than its top section. As a result, only a small part of optical energy penetrates through the slits. Localized SPPs at the sharp corners of the metallic nanostructures is relatively weak therefore cannot enable forward scattering and strong radiation modes. Meanwhile, one can see from Fig. 7(b) that the localized resonant waveguide modes are excited inside the sapphire substrate, which interfere destructively with incident light further suppressing radiation into the air side. So, the forward transmission is much larger than the backward transmission due to the different resonant interaction.

## Conclusions

In conclusion, we propose and numerically demonstrate a simple but elegant design composing of tapered metallic grating structure, which can exhibit high-contrast, broadband asymmetric transmission in the visible frequency range. The maximum transmission efficiency reaches ~95% for the forward illumination but only 35% for the backward illumination, and the corresponding transmission ratio is larger than 2.5 over a broadband wavelength regime. We investigated the effects of grating periodicity, metal thickness and substrate refractive index to fully understand the dependence of each parameter on the asymmetric transmission performance. The transmission window can be tuned by changing the design parameters. Our proposed design with high performance may have potential applications in compact and lightweight directional electromagnetic devices.

## Additional Information

**How to cite this article**: Tang, B. *et al*. Broadband asymmetric light transmission through tapered metallic gratings at visible frequencies. *Sci. Rep.* **6**, 39166; doi: 10.1038/srep39166 (2016).

**Publisher's note:** Springer Nature remains neutral with regard to jurisdictional claims in published maps and institutional affiliations.

## Supplementary Material

Supplementary Information
