# Supplementary material for: Broadband asymmetric light transmission through tapered metallic gratings at visible frequencies
Source: Sci Rep. 2016 Dec 13;6:39166. doi: 10.1038/srep39166 (PMC5153634; doi:10.1038/srep39166)
Supplement: Supplementary Information [file srep39166-s1.pdf]

Supplementary Information:

Broadband asymmetric light transmission through tapered metallic gratings at visible frequencies

Bin Tang[a,b], Zhongyang Li[b], Zizhuo Liu[b], Francois Callewaert[b], Koray Aydin[b,*]

[a] *School of Mathematics & Physics, Changzhou University, Changzhou 213164, China*

[b] *Department of Electrical Engineering and Computer Science, Northwestern University, IL 60208, USA*

* btang@cczu.edu.cn (B.T.); aydin@northwestern.edu (K.A.)

Figure S1

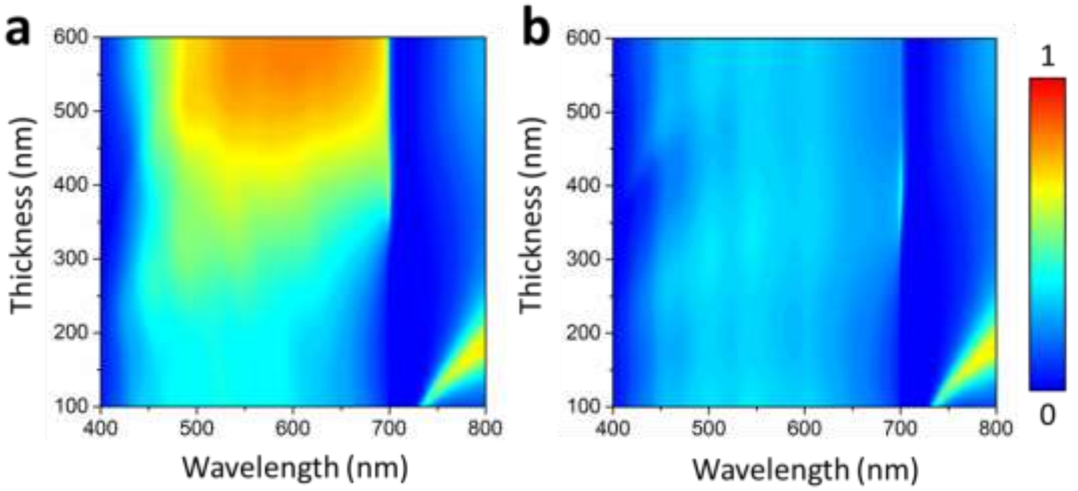

Figure S1. Bidirectional transmission spectra of light through an air-suspended TMGS versus wavelength for normal incidence and the thickness of TMGS with fixed periodicity p = 700 nm, and the other parameters keep the same as Figure 2 in text. (a) forward illumination, (b) backward illumination.

As a reference, Figure S1 depicted the bidirectional transmission as a function of wavelength for normal incidence and the grating thickness of air-suspended TMGS with fixed period p = 700 nm for forward and backward illumination, respectively. It

is clear that the metal thickness also plays a significant role in determining the asymmetric transmission behavior drastically. For thinner metallic gratings, there is no noticeable difference in transmission intensity between the forward and backward propagation. However, one can see that while the transmission intensity for forward propagation direction increases with increased metal thickness, for backward direction transmission intensity is barely affected. This observation suggests that thicker metallic grating arrays with tapered side profile have great potential for achieving asymmetric transmission.

Figure S2

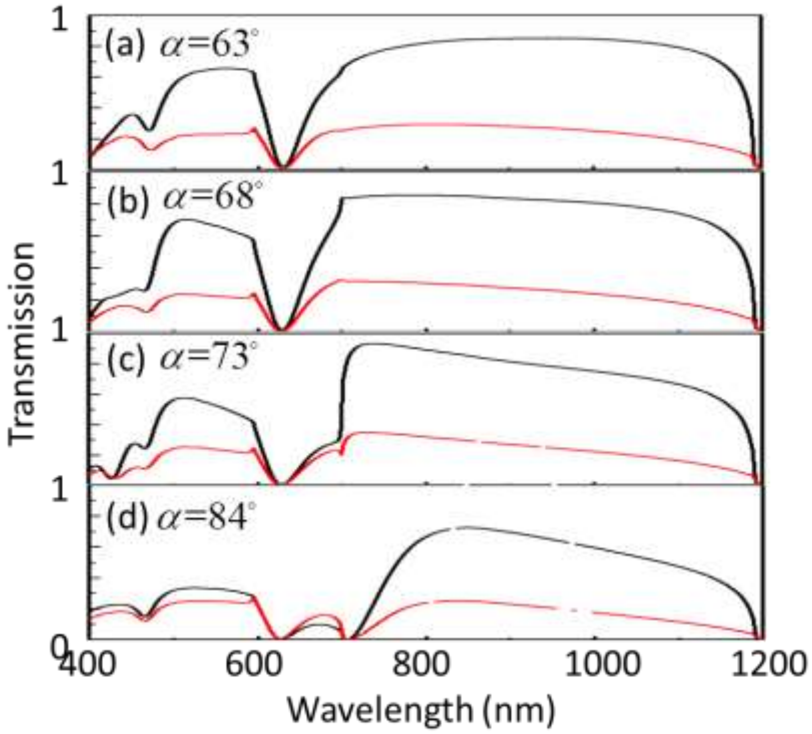

Figure S2. Bidirectional transmission spectra of light through different angle α in the presence of dielectric substrate. (a) $\alpha=63^{\circ}$,(b) $\alpha=68^{\circ}$, (c) $\alpha=73^{\circ}$, (d) $\alpha=84^{\circ}$. The other relevant parameters take the same as Figure 4 in the text.

Figure S2 illustrated the effect of angle α of TMGS on the transmission spectra for the forward and backward illumination. From this picture, one can see that the effect of angle cannot be omitted. When the angle takes a smaller value, the forward

transmission (denoted by black line) has a higher transmission than backward transmission, especially in the higher order mode bands. In other words, when the angle is gradually increased, the performance of asymmetric transmission would decrease correspondingly.

Figure S3

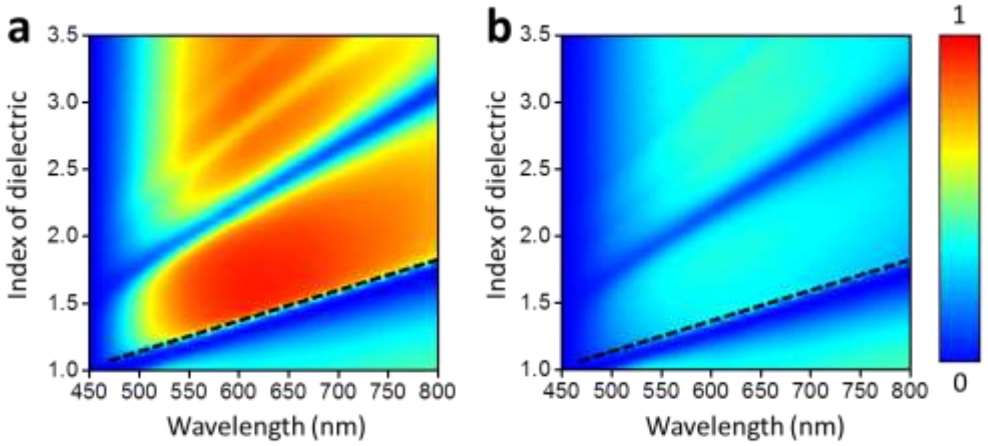

Figure S3. Transmission versus wavelength of normal illumination light and index of dielectric substrate for (a) forward illumination, and (b) backward illumination with fixed grating parameters *p*=440 nm, *b*=280 nm, and α=68 °.

In Figure S3, we made a further investigation of the BAT behavior for TM-polarized light illumination. It illustrates the total light transmission versus wavelength for normal incidence and index of dielectric substrate. From this Figure, one can see that the index of dielectric has an important effect on the BAT spectra. When the index of dielectric is smaller than 1.3, there is almost no asymmetric transmission. By contrast, when the index of dielectric takes a larger value than 1.3, there will appear an asymmetric transmission, and further enhancement of index can even induce a multiband asymmetric transmission due to the appearance of excited higher-order modes inside the dielectric. The black dot lines shown in Figure S3 represent the positions of Wood-Rayleigh anomalies, which determine the bandwidth of BAT band. Therefore, one can obtain BAT in the visible frequency by choosing optimized parameters and appropriate dielectric material. Note that this is for metal thickness of 120 nm. Higher metal thicknesses will still enable asymmetric transmission even there is no substrate.
